# Persistent human bocavirus 1 infection and tonsillar immune responses

**DOI:** 10.1002/clt2.12030

**Published:** 2021-08-12

**Authors:** Lotta E. Ivaska, Antti Silvoniemi, Oscar Palomares, Riitta Turunen, Matti Waris, Emilia Mikola, Tuomo Puhakka, Maria Söderlund‐Venermo, Mübeccel Akdis, Cezmi A. Akdis, Tuomas Jartti

**Affiliations:** ^1^ Department of Otorhinolaryngology – Head and Neck Surgery Turku University Hospital and University of Turku Turku Finland; ^2^ Swiss Institute of Allergy and Asthma Research University of Zürich Davos Switzerland; ^3^ Christine Kühne‐Center for Allergy Research and Education Davos Switzerland; ^4^ Department of Biochemistry and Molecular Biology School of Chemistry Complutense University of Madrid Madrid Spain; ^5^ Department of Pediatrics and Adolescent Medicine Turku University Hospital and University of Turku Turku Finland; ^6^ Children's Hospital Helsinki University Hospital and University of Helsinki Helsinki Finland; ^7^ Clinical Microbiology Turku University Hospital Turku Finland; ^8^ Institute of Biomedicine University of Turku Turku Finland; ^9^ Department of Otorhinolaryngology Satakunta Central Hospital Pori Finland; ^10^ Department of Virology University of Helsinki Helsinki Finland; ^11^ Department of Pediatrics Oulu University Hospital and University of Oulu Oulu Finland

**Keywords:** cytokine, forkheadbox protein 3, FOXP3, human bocavirus, RAR‐related orphan receptor C 2, RORC2, T‐helper_17_, T‐regulatory, tonsil, transcription factor

## Abstract

**Background:**

Persistent human bocavirus 1 (HBoV1) infection is a common finding in patients suffering from chronic tonsillar disease. However, the associations between HBoV1 infection and specific immune reactions are not completely known. We aimed to compare in vivo expression of T‐cell cytokines, transcription factors, and type I/III interferons in human tonsils between HBoV1‐positive and ‐negative tonsillectomy patients.

**Methods:**

Tonsil tissue samples, nasopharyngeal aspirate (NPA), and serum samples were obtained from 143 immunocompetent adult and child tonsillectomy patients. HBoV1 and 14 other respiratory viruses were detected in NPAs and tonsil tissues by polymerase chain reaction (PCR). Serology and semi‐quantitative PCR were used for diagnosing HBoV1 infections. Expression of 14 cytokines and transcription factors (IFN‐α, IFN‐β, IFN‐γ, IL‐10, IL‐13, IL‐17, IL‐28, IL‐29, IL‐37, TGF‐β, FOXP3, GATA3, RORC2, Tbet) was analyzed by quantitative reverse‐transcription (RT)‐PCR in tonsil tissues.

**Results:**

HBoV1 was detected by PCR in NPA and tonsils from 25 (17%) study patients. Serology results indicated prior nonacute infections in 81% of cases. Tonsillar cytokine responses were affected by HBoV1 infection. The suppression of two transcription factors, RORC2 and FOXP3, was associated with HBoV1 infection (*p* < 0.05). Furthermore, intratonsillar HBoV1‐DNA loads correlated negatively with IFN‐λ family cytokines and IL‐13.

**Conclusions:**

Our study shows distinctively decreased T‐helper_17_ and T‐regulatory type immune responses in local lymphoid tissue in HBoV1‐positive tonsillectomy patients. HBoV1 may act as a suppressive immune modulator.

## 1 BACKGROUND

Human bocavirus 1 (HBoV1) is a non‐enveloped single‐stranded DNA virus belonging to the Parvoviridae family.^1^ It was originally discovered in 2005 in respiratory‐tract specimens.^2^ HBoV1 is typically found in such specimens from young children suffering with lower respiratory‐tract infections such as bronchiolitis, wheezing, asthma, or pneumonia.^1^, ^3^ The diagnosis of acute bocavirus infection is based on quantitative polymerase chain reaction (PCR), expression of HBoV1 messenger RNA (mRNA), or serology, as the virus DNA may persist for weeks, even months.^1^, ^3^, ^4^, ^5^, ^6^ Interestingly, a few studies have found HBoV1 DNA in tonsil squamous cell carcinoma tumors, inducing speculations of a possible causal association.^7^, ^8^

Tonsils in the oropharynx are in contact with inhaled and ingested allergens and pathogens. Viral infection detected by PCR is a common finding in tonsil tissue of routine tonsillectomy patients.^9^ The prevalence of HBoV1 DNA in fairly asymptomatic tonsillectomy patients suffering chronic tonsillar disease has been 4%–27% in the tonsil tissue and 11%–18% in the nasopharyngeal aspirate (NPA) samples.^6^, ^9^, ^10^, ^11^, ^12^, ^13^ In our earlier study, the tonsillar cytokine expressions were associated with viral infections in general and clinical characteristics.^14^ Moreover, HBoV1 has been shown to modulate rhinovirus‐induced cytokine responses in wheezing children.^15^ Persistent viral infections of the tonsil tissue seem to alter the immune response, but the specific role of HBoV1 in chronic tonsillar disease remains unclear.

Human tonsils serve as an in vivo model for investigating cytokine responses at the local lymphoid tissue level. However, limited data are available on the bocavirus‐associated T‐cell and innate immune responses^16^ and immunologic effects overall. The investigation of these events is important as earlier studies suggest that HBoV1 may have immunomodulatory effects.^15^, ^17^ Therefore, our objective was to compare in vivo expression of T‐cell cytokines, transcription factors, and type I/III interferons in human tonsils between HBoV1‐positive and ‐negative, relatively healthy tonsillectomy patients. We hypothesized that HBoV1 infection may have immune suppressive activity.

## 2 METHODS

### 2.1 Study patients

Tonsil and NPA samples were collected from 200 adeno‐/tonsillectomy patients at Satakunta Central Hospital, Pori, Finland, between April 2008 and March 2009. Inclusion criteria were adenotomy, adenotonsillectomy, or tonsillectomy, according to clinical indication and written informed approval from the study subject or his/her guardian.^14^ The study protocols were approved by the Ethics Committee of the Satakunta Central Hospital and by the Ethics Committee of the Hospital District of Southwest Finland.

### 2.2 Sample collection

The internal part of the tonsil tissue removed from the patient was instantly cut into 3–4 mm cubes and stored in RNAlater (Qiagen, Hilden, Germany), first at +2 to 8°C until the next working day, and then at −80°C.^14^ NPA samples were collected through a nostril using a standardized procedure.^18^ If the aspirate yield was small, the collection was repeated after administering 2 ml of physiologic saline into a nostril. Peripheral blood samples were drawn for serology and allergy tests. The first of the paired serum samples was collected during the tonsillectomy anaesthesia and the follow‐up sample was taken after a median of 58 days (range 36–104). For viral analyses, a piece of the removed tonsils and the NPA were stored at −80°C.^14^ Study patients completed a standard questionnaire to collect information on health, medication, and respiratory symptoms within 30 days before the operation.^14^

### 2.3 Study outcomes

The aim of the study was to compare tonsil cytokine and transcription factor levels of IFN‐α, IFN‐β, IFN‐γ, IL‐10, IL‐13, IL‐17, IL‐28, IL‐29, IL‐37, TGF‐β, FOXP3, GATA3, RORC2, and Tbet between the HBoV1 DNA‐positive and ‐negative groups.

### 2.4 Virus diagnostics

Respiratory viruses in NPA and tonsil tissue samples were detected by PCRs (including the reverse transcription step when applicable) on nucleic acid extracts.^18^, ^19^ In‐house real‐time PCRs were used as described for HBoV1, enterovirus, rhinovirus, and respiratory syncytial virus.^20^, ^21^, ^22^ Primer sequences of HBoV1 have been previously described.^9^ Seeplex RV12 ACE Detection (Seegene, Seoul, Korea) multiplex PCR assay was applied for adenovirus, coronaviruses (229 E/NL63 and OC43/HKU1), influenza A and B viruses, metapneumovirus, parainfluenza virus types 1–3, respiratory syncytial virus groups A and B, and rhinovirus, according to manufacturer’s instructions. Quantitative PCR (qPCR) was used to measure tonsil HBoV1 DNA load (copies/g).^23^ Serological tests for HBoV1‐specific IgM and IgG were performed for 123 patients.^5^, ^23^ To verify that the IgG results were HBoV1 specific, the serum samples were blocked with HBoV2 and HBoV3 antigens. PCR tests were done at the Department of Virology, University of Turku, Finland. Serology was analyzed at the Department of Virology, University of Helsinki, Finland.

### 2.5 Cytokine analysis

Tonsil tissue was stabilized and then homogenized in grinding tubes containing CK28 ceramic beads by a Precellys 24 homogenizer (Bertin Technologies) two times at 6000 rpm for 50 s.^14^ An RNeasy mini kit (Qiagen, Hilden, Germany) was used to isolate total RNA from cell samples. Reverse transcription was performed with the Revert Aid M‐MuLV Reverse transcriptase (Fermentas, St. Leon‐Rot, Germany) according to the manufacturer’s protocol. Gene expression of IFN (interferon) ‐α, IFN‐β, IFN‐ɣ, IL (interleukin) ‐10, IL‐13, IL‐17, IL‐28, IL‐29, IL‐37, TGF‐β (tumor growth factor β), FOXP3 (forkhead box protein 3), GATA3 (GATA‐binding factor 3), RORC2 (RAR‐related orphan receptor C 2) and Tbet (T‐box transcription factor) were calculated by quantitative real‐time PCR using iTaq SYBR Green Supermix with ROX (Bio‐Rad, Hercules) on a 7900HT Fast Real‐Time PCR instrument (Applied Biosystems). Normalization was done by using housekeeping elongation factor 1α (EF1α). Data are presented as relative expressions, which show 2‐(ΔCT) values multiplied by 104, where ΔCT corresponds to the difference between EF1α and the CT value for the gene of interest.^10^, ^14^

### 2.6 Statistical analysis

Data were analyzed using SPSS software Statistics for Windows, version 26.0 (IBM SPSS Statistics for Macintosh). Continuous variables are described as medians and were analyzed using Mann‐Whitney *U* test due to skewed distribution. Categorical variables are expressed as frequencies and percentages and were analyzed by using Chi square test or Fisher’s exact test (when counts <5). Spearman correlations between virus load and cytokine levels and between transcription factors (RORC and FOXP3) and type 3 interferons were computed. Before regression analyses, cytokine and transcription factor values were log‐transformed because of positively skewed distributions of the data. Clinical, viral, and immunological differences between study groups were analyzed using unadjusted and multivariable linear model analysis. The adjustments for immunologic analyses included clinical factors and virus infections, which significantly differed between the groups, and age. The backward stepwise method was used for the final adjustment model separately for each cytokine and transcription factor. Only statistically significant factors were kept in the model. Statistical significance was established at the level of *p *< 0.05.

## 3 RESULTS

### 3.1 Study population

Initially, enrolling involved 200 patients, of whom 45 were sole adenotomy patients and 12 had otherwise insufficient sample material. Thus, 143 suitable tonsil (right or left) and NPA samples from tonsillectomy patients were analyzed. The excluded patients did not differ from the included patients by age, sex, or indication for tonsillectomy.

### 3.2 Patient characteristics

HBoV1 DNA in NPA and tonsil tissue was detected by PCR in 25/143 (17%) patients with a median age of 6 years; see Tables 1 and 3. The median age of HBoV1‐negative patients (118/143%, 83%) was 18 years (*p* < 0.001). The sex distribution between the study groups was equal; 56% were male in the HBoV1‐positive group and 52% in the ‐negative group. There was no difference in self‐reported allergy, sensitization, allergic diseases, active/passive smoking, or symptoms on the operation day between the groups. Tonsillar hypertrophy was a more common indication for the operation in the HBoV1‐positive group (16%, 64%) compared to the HBoV1‐negative group (32%, 27%) (*p* < 0.001). In contrast, recurrent tonsillitis was a more common indication in the HBoV1‐negative group compared to the ‐positive group (40%, 34% vs. 3%, 12%) (*p* = 0.03). See more patient characteristics in Table 1.

**TABLE 1:** Patient characteristics of tonsillectomy patients (*n* = 143) divided in HBoV1 DNA‐positive and ‐negative NPA or tonsil tissues

Factor	HBoV1‐DNA positive, *n* = 25	HBoV1‐DNA negative, *n* = 118	*p*‐value
Median age (range), years	6.1 (2–22)	18.3 (2–65)	<0.001
Male	14 (56%)	61 (52%)	0.83
Indication for adeno‐/tonsillectomy			
Recurrent tonsillitis	3 (12%)	40 (34%)	0.03
Tonsillar hypertrophy	16 (64%)	32 (27%)	<0.001
Other indication^a^	1 (4%)	5 (4%)	1.00
Mixed indication of these	5 (20%)	41 (35%)	0.52
Self‐reported allergy	14/24 (58%)	49/103 (48%)	0.34
Sensitization	9/20 (45%)	41/96 (43%)	0.85
Food	4	30	
Aero (i.e., pollen, animal, or house dust mite)	4	4	
Both food and aero	1	7	
Physician‐diagnosed asthma	3/17 (18%)	9/82 (11%)	0.44
Self‐reported allergic rhinitis	5/17 (29%)	22/83 (27%)	0.80
Physician‐diagnosed atopic dermatitis	2/18 (11%)	12/84 (14%)	0.77
Smoking or exposure to smoking	12/23 (52%)	65/113 (58%)	0.64
Respiratory symptoms on the operation day^b^	4/20 (20%)^c^	20/106 (19%)	0.91
Respiratory symptoms within 2 weeks prior to the operation day^b^	11/20 (55%)	38/97 (39%)	0.19

*Note:* Data are expressed as number of subjects (%) except age. Differences between atopic versus nonatopic subjects were calculated with Mann Whitney *U* test, Chi square test, or Fischer exact test (when counts <5).

^a^ Chronic white patches in tonsils (*n* = 2), recurrent fever (*n* = 1), throat abscess (*n* = 1), teeth braces (*n* = 1), and food remnants in tonsils (*n* = 1).

^b^ One or more of the following: mild rhinitis, cough, symptoms of otitis, throat pain, upper airway obstruction symptoms.

^c^ HBoV1 DNA‐positive: two tonsil tissues, one NPA, one tonsil and NPA.

### 3.3 Virus findings

HBoV1 was detected in 18 (13%) of the NPA samples and in 12 (8%) of the tonsil tissue samples. In five patients, the virus was found in both sample types. HBoV serology was analyzed from 123 of the 143 (86%) tonsillectomy patients. Furthermore, paired serum samples were collected from 50/123 (41%) patients. Seroconversion of HBoV1 IgG was detected in one case and IgG positivity was detected in 100/123 (81%) of the cases. In 18 cases, the positive HBoV1‐DNA finding was accompanied by a positive‐IgG and negative‐IgM result indicating a prior nonacute infection. Furthermore, 3/123 (2%) were HBoV1 IgM positive and one HBoV2‐IgM positive. See Table 2 for more information. Concerning all samples (NPA and tonsils) together, the most common co‐infection with HBoV1 was rhinovirus, with 19 in the NPA samples and 3 in the tonsil samples. In the HBoV1‐negative group, the most common viral finding was rhinovirus, with 48 in the NPA samples and 3 in the tonsil samples. In the tonsil samples, the most common virus finding was HBoV1 (12 cases). The second most common viruses were adenovirus and enterovirus (seven cases of each). See detailed virus findings in Table 3.

**TABLE 2:** Human bocavirus serology findings

Human bocavirus serology	*n* = 123	
HBoV1, ‐2, or ‐3 IgG	122	99%
HBoV1 IgG	100	81%
HBoV1, ‐2, or ‐3 IgG seroconversion	1	1%
HBoV1 IgG seroconversion	1	1%
HBoV1, ‐2, or ‐3 IgM	4	3%
HBoV1 IgM	3	2%
HBoV2 IgM	1	1%
HBoV3 IgM	0	0%
Total acute HBoV serodiagnosis	4	3%

*Note:* One case was barely above cut‐off for HBoV1 IgM and intratonsillar HBoV1‐PCR positive.

**TABLE 3:** Virus nucleic acid detection in the nasopharynx or tonsils

Virus	HBoV1‐DNA pos *n* = 25	HBoV1‐DNA neg *n* = 118	*p*‐value
Nasopharynx			
Rhinovirus	19 (76%)	49 (42%)	*0.002*
Human bocavirus 1	18 (72%)	‐	‐
Adenovirus	8 (32%)	6 (5%)	*<0.001*
Enterovirus	3 (12%)	8 (7%)	0.41
Coronavirus^a^	2 (8%)	7 (6%)	0.66
Metapneumovirus	1 (4%)	1 (1%)	0.32
Respiratory syncytial virus	1 (4%)	1 (1%)	0.32
Influenza A or B virus	0 (0%)	2 (2%)	1.0
Parainfluenza virus types 1–3	0 (0%)	3 (2%)	1.0
Two or more viruses	18 (72%)	11 (9%)	*<0.001*
Tonsil			
Human bocavirus 1	12 (48%)	‐	‐
Adenovirus	5 (20%)	7 (6%)	*0.037*
Enterovirus	5 (20%)	7 (6%)	*0.037*
Rhinovirus	3 (12%)	3 (2.5%)	0.066
Parainfluenza virus types 1–3	2 (8%)	5 (4%)	0.35
Respiratory syncytial virus	2 (8%)	0 (0%)	*0.03*
Metapneumovirus	1 (4%)	1 (1%)	0.32
Coronavirus^a^	0 (0%)	1 (1%)	1.0
Influenza A or B virus	0	0	‐
Two or more viruses	8 (32%)	3 (2.5%)	*<0.001*
Nasopharynx and tonsil			
Human bocavirus 1	5 (20%)	‐	‐
Enterovirus	1 (4%)	4 (3%)	1.0
Rhinovirus	3 (12%)	1 (1%)	*0.017*
Adenovirus	2 (8%)	0	*0.03*
Coronavirus^a^	0	1 (1%)	1.0
Influenza A or B virus	0	0	‐
Metapneumovirus	0	0	‐
Parainfluenza virus types 1–3	0	0	‐
Respiratory syncytial virus	0	0	‐
Two or more viruses	16 (64%)	12 (10%)	*<0.001*

*Note:* Data are expressed as number of subjects (%). Differences between HBoV1 positive versus HBoV1 negative subjects were calculated with Chi square test or Fischer exact test (when counts <5).

^a^ Coronaviruses 229E, OC43, NL63, and HKU1.

### 3.4 Cytokine and transcription factor findings

**FIGURE 1:**
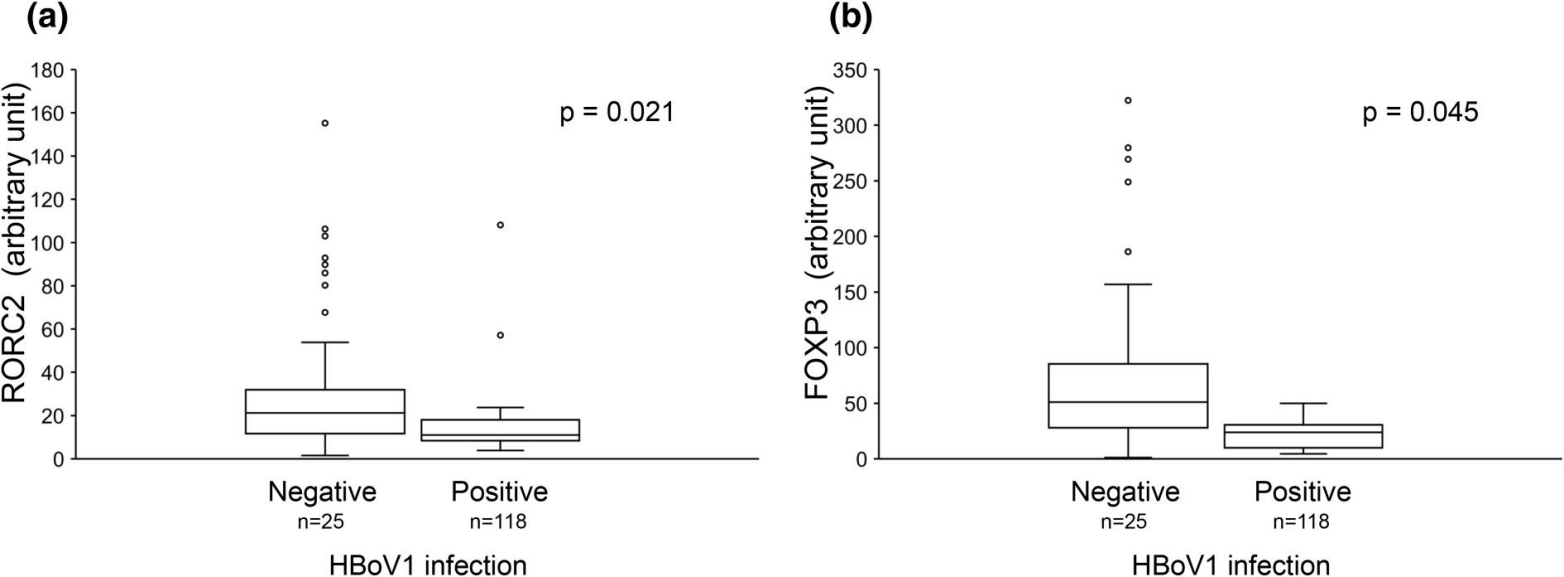
Relative tonsillar expression of forkhead box protein 3 (FOXP3) and RAR‐related orphan receptor C 2 (RORC2). Comparison of tonsil samples of patients with human bocavirus 1 (HBoV1) DNA‐negative or ‐positive NPA or tonsils. Values are arbitrary units × 10^4^ relative to EF1α

**FIGURE 2:**
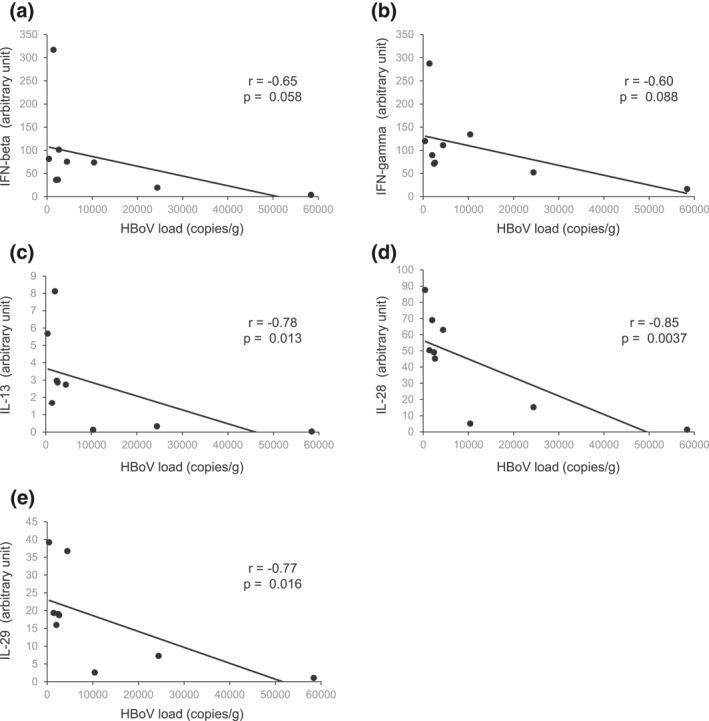
(A–E) Correlations between intratonsillar HBoV1‐DNA loads and cytokine expressions. The HBoV1 DNA load (*x*‐axis) is presented as copies/g and the cytokine expressions (*y*‐axis) as arbitrary units × 10^4^ relative to EF1α

The cytokine and transcription factor findings were different in HBoV1‐positive and ‐negative patients. After univariate analysis, the expressions of T‐helper_17_ (Th17) type transcription factor RORC2 and T‐regulatory type transcription factor FOXP3 were significantly lower in the HBoV1‐positive group (*p* = 0.021, *p* = 0.045); see Figure 1. The results persisted in multivariable analysis after adjusting for differences between the groups; see Table 4. Comparing sole HBoV1 DNA‐positive (*n* = 12) and ‐negative (*n* = 131) tonsil samples, RORC2 and FOXP3 expression levels remained lower in the sole HBoV1‐positive group (both, *p* < 0.05). Moreover, there was no difference between the two groups when comparing IL‐28, IL‐29, and IL‐13, but the tonsil HBoV1‐DNA load correlated negatively with IL‐28 (*p* = 0.004), IL‐29 (0.019), and IL‐13 *(p* = 0.013) and tended to correlate negatively with IFN‐β (*p* = 0.058) and IFN‐γ (*p* = 0.088). Age had no effect on the DNA load correlation (Figure 2). There was no correlation between RORC2 and FOXP3 with Type 3 interferons in HBoV1 positive patients (data not shown).

**TABLE 4:** Cytokine or transcription factor expression in tonsils of patients with HBoV1 DNA‐positive or ‐negative NPA or tonsil tissue

Cytokine or transcription factor	HBoV1 positive *n* = 25	HBoV1 negative *n* = 118	*p* value univariate	*p* value multivariate	Adjustments
T‐h1‐type					
IFN‐γ	65 (35, 104)	65^a^ (34, 103)	0.96	0.58	Rhinovirus NPA and tonsil
Tbet	54 (22, 79)	45 (20, 71)	0.65	0.14	Age, rhinovirus NPA, and tonsil
T‐2‐type					
IL‐13	1.4 (0.3, 3.2)	0.5 (0.02, 4.1)	0.19		‐
GATA3	24 (10, 31)	24 (11, 39)	0.27		‐
T‐helper17‐type					
IL‐17	10 (7, 19)	10 (5, 18)	0.18	0.47	Age, RSV tonsil
RORC2	11 (7, 19)	21 (11, 32)	*0.021*		‐
T‐reg‐type					
IL‐10	57 (25, 73)	42 (23, 67)	0.20	0.55	Age
IL‐37	0.24 (0.14, 0.35)	0.17^c^ (0.11, 0.34)	0.10	0.91	Age
FOXP3	35 (14, 60)	51 (28, 86)	*0.045*		‐
TGF‐β	140 (104, 234)	163 (105, 217)	0.89		‐
Type I/III interferons					
IFN‐α	20 (1, 181)	10^b^ (0.4, 128)	0.23		‐
IFN‐β	37 (7, 154)	16^a^ (3, 100)	0.23		‐
IL‐28	40 (8, 78)	17^a^ (2, 76)	0.11		‐
IL‐29	16 (3, 36)	6 (2, 28)	0.59		‐

*Note:* Values are arbitrary units × 10^4^ relative to EF1α. Data are expressed as median (interquartile range). Adjustments are selected backward stepwise from significant differences between groups (age, recurrent tonsillitis, tonsillar hypertrophy, rhinovirus in nasopharyngeal aspirate (NPA), adenovirus in NPA, tonsillar adenovirus, tonsillar enterovirus, tonsillar respiratory syncytial virus (RSV), adenovirus in tonsil and NPA, rhinovirus in tonsil and NPA, two or more viruses in NPA, tonsil or both).

Abbreviations: FOXP, forkhead box protein; GATA3, GATA‐binding factor 3; IFN, interferon; IL, interleukin; RORC, RAR‐related orphan receptor C; Tbet, T‐box transcription factor; TGF, tumor growth factor; Th, T helper cell; Treg, T regulatory cell.

^a^ *n* = 117.

^b^ *n* = 116.

^c^ *n* = 115.

## 4 DISCUSSION

This study provides new information about tonsillar cytokine and transcription factor responses in persistent HBoV1 infection. Our study shows three important findings. First, persistent HBoV1 is common (17%) in NPA and tonsil samples of tonsillectomy patients. Second, persistent HBoV1 infection is associated with suppression of cytokine and transcription factor responses in tonsil tissue. This is especially seen within the important transcription factors of innate immunity and immune regulation, RORC2 and FOXP3. Third, the intratonsillar HBoV1‐DNA load correlates negatively with IL‐28, IL‐29, and IL‐13. In our knowledge, this is the first study to compare tonsillar cytokine responses in vivo between HBoV1 DNA‐positive and ‐negative patients using NPA and/or tonsil samples.

We studied tonsillectomy patients who had no severe respiratory symptoms during the operation day. HBoV1 DNA was detected by PCR in tonsil and/or NPA samples in 17% of the study patients; the prevalence in the tonsil tissue samples was 8%. This is in line with earlier studies.^9^, ^12^ Furthermore, HBoV infections are most prevalent in small children, showing prolonged virus shedding in the nasopharynx after acute infection, whereas they become less common with age.^24^ The overall HBoV1 IgG seroprevalence was 81%, and only one tonsillar HBoV1 DNA‐positive case was serologically identified as a putative recent infection by HBoV1‐IgM positivity. This finding reflects prolonged persistence as demonstrated earlier.^1^, ^3^, ^12^ Consequently, HBoV1 is a common finding in children suffering from chronic adenotonsillar disease.

RORC2 is the master transcription factor for T‐helper_17_ cell generation.^25^, ^26^ Th17 cells are derived from naive T‐cells by the activation of antigen presenting cells, differentiation cytokines, and transcription factor RORC2. Th17 cells are the major source for IL‐17 production. IL‐17 A and IL‐17F activate cells in various tissues to produce proinflammatory cytokines, chemokines, and metalloproteases. Activation of these cell‐mediating proteins results in inflammatory processes, immune responses to extracellular pathogens and autoimmunity. Overall, IL‐17A and IL‐17F are associated with, for example, rheumatoid arthritis, inflammatory bowel disease, psoriasis, allergic asthma, and atopic dermatitis.^25^, ^26^ In our study, the expression of RORC2 in the tonsil tissue was lower within the HBoV1‐positive group. This finding indicates that HBoV1 is associated with Th17‐type cytokine reactions by affecting the transcription factor RORC2. However, there was no difference in the expression levels of IL‐17 between HBoV1‐positive and ‐negative groups. This might indicate that part of IL‐17 production occurs in NK cells, neutrophils, CD8^+^ T cells, and γð T cells.^25^ It has been shown earlier that co‐infection of HBoV1 and rhinovirus results in a modified non‐Th2‐type response, which is different than the cytokine response of either virus alone.^15^ RORC2 has been investigated recently concerning inflammatory bowel diseases. Inhibition of RORC2 on in vivo‐differentiated T‐cells suppressed pathogenic IL‐17A production and increased anti‐inflammatory IL‐10 production.^27^ This finding shows the important role of RORC2 in innate immunity.

FOXP3 acts as a transcription factor for regulatory T‐cells (T‐reg) differentiating from naive T‐cells after antigen stimulation. Differentiation into functioning T‐reg cells requires the presence of TGF‐ß and IL‐2 and transcription factor FOXP3. Certain T‐reg cells produce anti‐inflammatory cytokines such as IL‐10 and TGF‐ß. The function of IL‐10 is immunosuppressive and it is particularly associated with allergic diseases. TGF‐ß plays a role in embryonal tissue formation and influences the balance of pro‐ and anti‐inflammatory cytokines. TGF‐ß is strongly associated with autoimmune diseases, Alzheimer disease, cardiovascular pathologies, cancer, allergic rhinitis, and fibrosis.^25^ FOXP3+ T‐reg cell function is highly studied due to the potential in clinical practice.^28^ It has been demonstrated that the frequency of FOXP3+ T‐reg cells in tonsil tissue is three times higher than in peripheral blood.^29^ Interestingly, in our study, the expression of T‐reg transcription factor FOXP3 was lower in the tonsil tissues of patients in the HBoV1 DNA‐positive group.

Furthermore, our study shows a negative correlation between intratonsillar HBoV1‐DNA loads and IL‐28, IL‐29 (IFN‐λ family). The secretion of IL‐28 and IL‐29 and other IFNs is an important defence mechanism against respiratory viral infections.^25^ It has been shown that IFN‐λ provides antiviral defence especially at the epithelium surface of the respiratory tract.^30^ Contoli et al. showed a clear inverse correlation between rhinovirus load and IFN‐λ production in bronchial epithelial cells.^31^ HBoV1 may also reduce IFN responses as shown in vitro.^17^ Our study is the first one that compares IFN‐λ and HBoV1‐DNA loads.

Moreover, the correlation between intratonsillar HBoV1‐DNA loads and IL‐13 was also negative. IL‐13 is part of T‐helper_2_ type immune response and is especially associated with allergic diseases such as asthma and rhinitis.^25^ Interestingly, a recent study shows an increase of type 2 immune effectors including IL‐13 in severe COVID‐19 compared to a moderate one.^32^ IL‐13 seems to relate to virus‐induced immune reactions. Thus, low IFN and IL‐13 expressions could promote persistence of viral infections. In earlier study, HBoV has been associated with upregulation of type 2 immune response in bronchoalveolar lavage fluid. Especially cytokines linked with lung fibrosis and tumor development were expressed, prompting speculations of possible causal correlation.^33^ Furthermore, this finding supports the role of HBoV as an immunomodulator.

The strengths of this study are the direct analyses of tonsil tissue samples and the thorough characterization of the study subjects. Additionally, persistent HBoV1 infection was carefully diagnosed by PCR and serology. The limitations of the study include a relatively small number of study subjects (all *n* = 143, HBoV1 positive *n* = 25) and that the data set was not complete. HBoV1‐DNA loads were measured from 9 of the 12 HBoV1 positive tonsil tissue samples. Serum samples were obtained from 123 cases. Due to ethical reasons, we did not study a healthy control group.

## 5 CONCLUSION

In conclusion, our study shows that persistent HBoV1 infection is associated with suppression of tonsillar cytokine responses, especially transcription factors RORC2 and FOXP3. This finding supports the earlier study^15^ that HBoV1 infection may have immunosuppressive capacity and warrants further study.

## AUTHOR CONTRIBUTIONS

The study protocol and manuscript were written by the investigators. Data were collected by Tuomo Puhakka, Lotta E. Ivaska, and Emilia Mikola and analyzed by Lotta E. Ivaska, Antti Silvoniemi, and Tuomas Jartti. Immunologic analyses were done by Oscar Palomares and Riitta Turunen and supervised by Mübeccel Akdis and Cezmi A. Akdis. Viral analyses were carried out by Matti Waris. Serology was analyzed by Maria Söderlund‐Venermo. The study was supervised by Cezmi A. Akdis and Tuomas Jartti. The granting agencies covered all costs and played no role in study design, data analysis, or manuscript preparation. The first draft was written by Lotta E. Ivaska. All authors read and approved the final manuscript.
